# Acceptability of wearable technology for the early detection of dementia-causing diseases: perspectives from the CODEC II cohort

**DOI:** 10.1186/s44247-025-00191-3

**Published:** 2025-08-29

**Authors:** Sarah Wilson, Emily Beswick, Rachel Morrell, Sharandeep Bhogal, Clare Tolley, Tim Whitfield, Kieran Wing, Riona Mc Ardle, Nehal Hassan, Zuzana Walker, Sarah Slight

**Affiliations:** 1 School of Pharmacy, Newcastle University, King George VI Building, Queen Victoria Road, Newcastle Upon Tyne, UK; 2 Trinity College Dublin, Dublin, Ireland; 3 University College London, London, UK; 4 Essex Partnership University NHS Foundation Trust, Essex, UK; 5 Translational and Clinical Research Institute, Newcastle University, Newcastle Upon Tyne, UK

**Keywords:** Qualitative, Dementia, Early detection, Digital health technologies, Wearable technologies

## Abstract

**Background:**

The global prevalence of dementia is increasing exponentially. Early detection of dementia-causing diseases could support therapeutic intervention to decelerate disease progression. Wearable digital technologies can be used to identify early signs of such diseases and remotely monitor disease progression. However, technologies must be acceptable to users. This study explored the perspectives of participants on the acceptability of various wearable technologies for early detection.

**Method:**

Participants from the Cognitive Decline using Digital Devices (CODEC-II) cohort used four different wearables (smartwatch, electroencephalographic [EEG] headband, active and passive smartphone apps) for two weeks every three months over a year. A subgroup participated in semi structured interviews after two weeks to discuss their experiences and acceptance of the devices. Data was analysed using the framework analysis approach, aided by N-Vivo (v14.23.2).

**Results:**

Twenty-one participants were interviewed, including individuals with subjective cognitive decline (*n* = 10), mild cognitive impairment (MCI) (*n* = 7), dementia with Lewy bodies (*n* = 1), and three caregivers. Five key themes were identified, including ease of use, wearability, usefulness, transparency, and behavioral intention. Many participants relied on the research team to help set up the technology for them due to high levels of digital anxiety. Individuals with MCI particularly struggled with cognitive testing games in the active smartphone app, which they experienced increased awareness of their own cognitive impairments. Participants preferred wrist-worn over head-worn devices due to familiarity and impact on their appearance. While some participants enjoyed using the wearables, others questioned their accuracy for medical purposes. There was also a lack of understanding around what data were being collected from the wearables and how it was being collected, with some expressing concerns about data disclosure. Participants with professional or caregiving responsibilities described how their busy lifestyles hindered daily use of the wearables.

**Conclusion:**

These findings highlight the importance of using an inclusive design approach to meet users’ needs and support digital inclusivity, and an effective communication strategy to increase transparency and reduce data disclosure concerns. Future research is needed to explore the effectiveness of implementing current recommendations to support digital health equity and codesign a communication strategy with users to ensure the information is understandable.

**Trial registration:**

CODEC-II was retrospectively registered as a clinical trial under the registry ClinicalTrials.gov (trial registration number: NCT07051408, date of registration: 3rd June 2025).

**Supplementary Information:**

The online version contains supplementary material available at 10.1186/s44247-025-00191-3.

## Background

Global prevalence of dementia is estimated to reach 152.8 million by 2050. [1] Dementia is a syndrome associated with the deterioration of cognitive function beyond normal aging [2] and is caused by a variety of different diseases that destroy neurons and damage neurophysiological processes [3]. The most common type of dementia-causing disease is Alzheimer's disease (AD), a progressive neurodegenerative disorder characterized by neuropathologic findings of amyloid plaques and neurofibrillary, tau-based tangles [4]. These features may begin 15 to 20 years before obvious cognitive symptoms [5]. Detecting dementia-causing disease early could support therapeutic intervention to decelerate disease progression, such as Lecanemab, a pharmacological intervention recently approved for use in the United Kingdom (UK) for the early stages of Alzheimer’s disease [6]. Current dementia diagnostic workup includes a combination of expensive and invasive techniques, including neuroimaging and cerebral spinal fluid analysis, used once symptoms of dementia-causing diseases become clinically apparent [7, 8].

Researchers are currently exploring various other methods for detecting subtle changes in cognition, behavior and physiology early, such as the use of blood-based biomarkers (e.g., beta-amyloid) [9, 10]. These biomarkers have shown promise and could help differentiate between different dementia-causing diseases in the future [11]. Wearable technologies can also collect data and monitor changes in behavior, such as gait [12], sleep [13] and cognition [14]. These technologies can be worn on the body or attached to clothing and provide a complementary, inexpensive, minimally invasive, and scalable techniques to support early detection for research purposes [15]. They can also capture changes in real-life (i.e., outside of the clinic) behavior, which may offer advantages over traditional methods, such as pen and paper cognitive screening tests such as the General Practitioner Assessment of Cognition (GPCOG) [7].

Regulatory bodies, such as the European Medicines Agency (EMA), the United States Food and Drug Administration (FDA) and National Institute for Health and Care Excellence (NICE), have sought evidence of user involvement in design and development of digital technologies for clinical practice [16–18]. This includes seeking the perspectives of those most at risk of developing dementia-causing diseases, for example, people with subjective cognitive decline (SCD) (the self-reported experience of worsening cognitive function) [19, 20], and mild cognitive impairment (MCI) (an intermediate cognitive state between normal aging and dementia, although not everyone with MCI progresses to dementia) [21, 22]. It is also important to seek the views of those with a clinical diagnosis of dementia as their data, collected from wearables, could be used to monitor their disease progression; their caregivers are also likely be involved in supporting the use of this technology.

Previous research suggests specific features of technologies, such as aesthetics, can influence the acceptance of wearable technologies among people living with dementia and their caregivers [23]. Acceptability may also change with users’ experience with an intervention [24]. Thus, we aimed to explore the perspectives of those living with a clinical diagnosis of dementia, SCD and MCI and their caregivers on the acceptability of the use of four wearable technologies measuring modalities of physical activity, sleep and cognitive function.

## Method

### Study design

This study is part of the ‘Early Detection of Neurodegenerative Diseases Initiative’ (EDoN), an international consortium seeking to develop a clinically appropriate toolkit, composed of wearable technologies to support the early detection of dementia-causing diseases [15]. We conducted semi-structured qualitative interviews to explore the concurrent acceptability (a construct that reflects the extent to which an individual receiving an intervention finds it appropriate based on their cognitive and emotional responses while participating) [24] of wearables among older adults with cognitive impairments and caregivers to support the development of the EDoN toolkit.

### Participants

#### Recruitment and eligibility criteria

Participants were recruited through an existing EDoN affiliated research cohort, ‘predictors of COgnitive DECline using digital devices’ (CODEC-II) [25]. Participants in this cohort attended the Essex Memory Clinic at Essex Partnership University NHS Foundation Trust (Southeast England, UK) for either cognitive complaints or impairments or were relatives/caregivers of someone with cognitive complaints or impairments. The patient’s cognitive status had been previously determined/diagnosed by qualified healthcare professionals within the Trust’s memory clinic and was not measured as part of this research. Study eligibility criteria included the ability to speak English (to support comprehensive communication between participants and the research team who only spoke in English), owning a personal smartphone with internet access at home and having the ability to provide informed consent. Individuals who were diagnosed with an active major psychiatric disorder (e.g., schizophrenia, bipolar affective disorder), had a sensitivity or allergic reaction to latex, rubber or plastics (to minimize harm when using the wearable technologies), participating in any other clinical trials, and/or were receiving end-of-life care were excluded.

During the CODEC-II recruitment process, researchers shared information about this qualitative sub-study in person both verbally and in a written form (via an information sheet) to all (n = 100) who were enrolled in the cohort. We did not record the reasons why individuals declined to participate. EB or SW contacted participants who volunteered to take part to organize a remote interview (phone call or Zoom (Zoom Technologies, Inc.)) after written consent was provided. Verbal consent was reconfirmed at the beginning of the interview.

#### Procedure

**Fig. 1:**
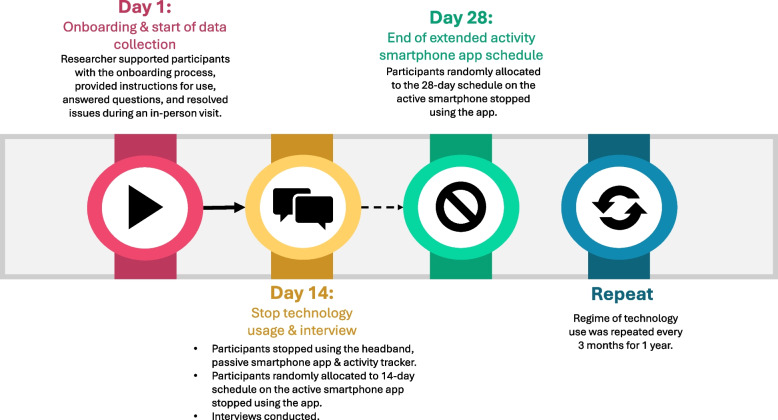
Summary of the research procedure

Participants used four wearable technologies that were selected based on a scoping review of commercially available technologies capable of measuring modalities associated with the early signs of dementia-causing diseases. This included a wrist-worn activity tracker, an electroencephalographic (EEG) headband, and a passive and active smartphone app (Table 1). Participants used the EEG headband, activity tracker and passive app for 14 days, but were randomly allocated to either a 28-day schedule or a 14-day schedule on the active smartphone (Table 1). This schedule was repeated every three months for one year (Fig. 1).

**Table 1:** Summary of the wearable technologies’ metrics and duration of use

Device	Position device is worn	Method of data collection	Behavioral and physiological measures	No. of consecutive days	Duration of engagement
Activity tracker	Wrist	Passive collection via a three-axis accelerometer and the optical heart rate sensor	• Activity levels (step count) • Sleep • Heart rate	14	Worn during the day and night
EEG* Headband	Head	Passive collection via 6 dry inbuilt EEG sensors	• Sleep	14 (nights)	Worn in bed at night
Passive smartphone app	Held in hand	Passive collection during normal smartphone use	Fine motor movements and reaction time to different tasks when using the touchscreen of their smartphone including: • Gestures used (swipes and taps) • Orientation of the smartphone (the way the phone is pointing) • Keystrokes with characters redacted	14	Normal smartphone usage
Active smartphone app	Held in hand	Gamified cognitive assessment and self-reported scales Participants were given one or two games (depending on the aspect of cognition being assessed) to complete per day on a random schedule	Different games assessed aspects of cognition including: • Episodic memory • Language • Executive function • Sematic processing • Visuospatial • Self-reported scales measured: • Mood • Sleep • Subjective cognitive function	14 or 28 depending on participants'random allocation	5–10 min per day

^*^EEG Electroencephalogram

A member of the research team (RM) (who had received training on how to set up and use the wearables by another member of the research team (SB) who worked closely with the technology companies and developers) provided each participant with the wearables and supported the initial on-boarding process during an in-person visit. This included helping participants download the relevant apps, create accounts, and pair the wearable devices with their personal smartphones. They also provided instructions on how to use the wearables, answered questions that they may have about the onboarding process, and resolved issues.

#### Data collection

Participants recruited for the CODEC-II study completed a quantitative questionnaire prior to taking part in the interview to gather relevant data on their sociodemographic background (e.g., date of birth, biological sex, ethnicity, educational attainment, residential type), medical history (e.g., self-reported diagnosis of dementia, MCI or SCD (including date of diagnosis) and other diseases/conditions/comorbidities)) and psychiatric history. Anonymized responses (e.g., birth year provided to calculate age) for those taking part in this qualitative sub-study were shared securely with the qualitative research team.

Semi-structured interviews were conducted at the end of participants’ first two weeks of using the technologies (between February 2023 and October 2023) to gather their initial perspectives and experiences. The interview topic guide was designed by the qualitative research team and explored key aspects relating to the research aims; it was piloted with nine individuals with various cognitive impairments, dementia, and their caregivers (Appendix 1) [26]. Open ended questions explored participants’ acceptance, barriers, and facilitators of using the different technologies. A researcher trained in qualitative methodologies (EB) conducted remote interviews (Zoom Technologies, Inc., or phone calls) lasting between 20 and 40 min. Interviews were audio recorded with permission, transcribed verbatim by a transcription company (University Transcriptions), and then anonymised and checked by the research team for accuracy. Participant names were replaced with a participant ID that included a number (chronological order of interviews), participant’s cognitive status and gender (e.g., P1, F, person with SCD = participant who was interviewed first, identified as female and had subjective cognitive decline).

### Data analysis

#### Quantitative questionnaire

The quantitative questionnaire was analyzed via descriptive statistics using Excel (Microsoft, version 2412) to provide an overview of participants’ age, gender, ethnicity, educational attainment, and date of diagnosis or caregivers (no cognitive impairment).

#### Qualitative interviews

**Fig. 2:**
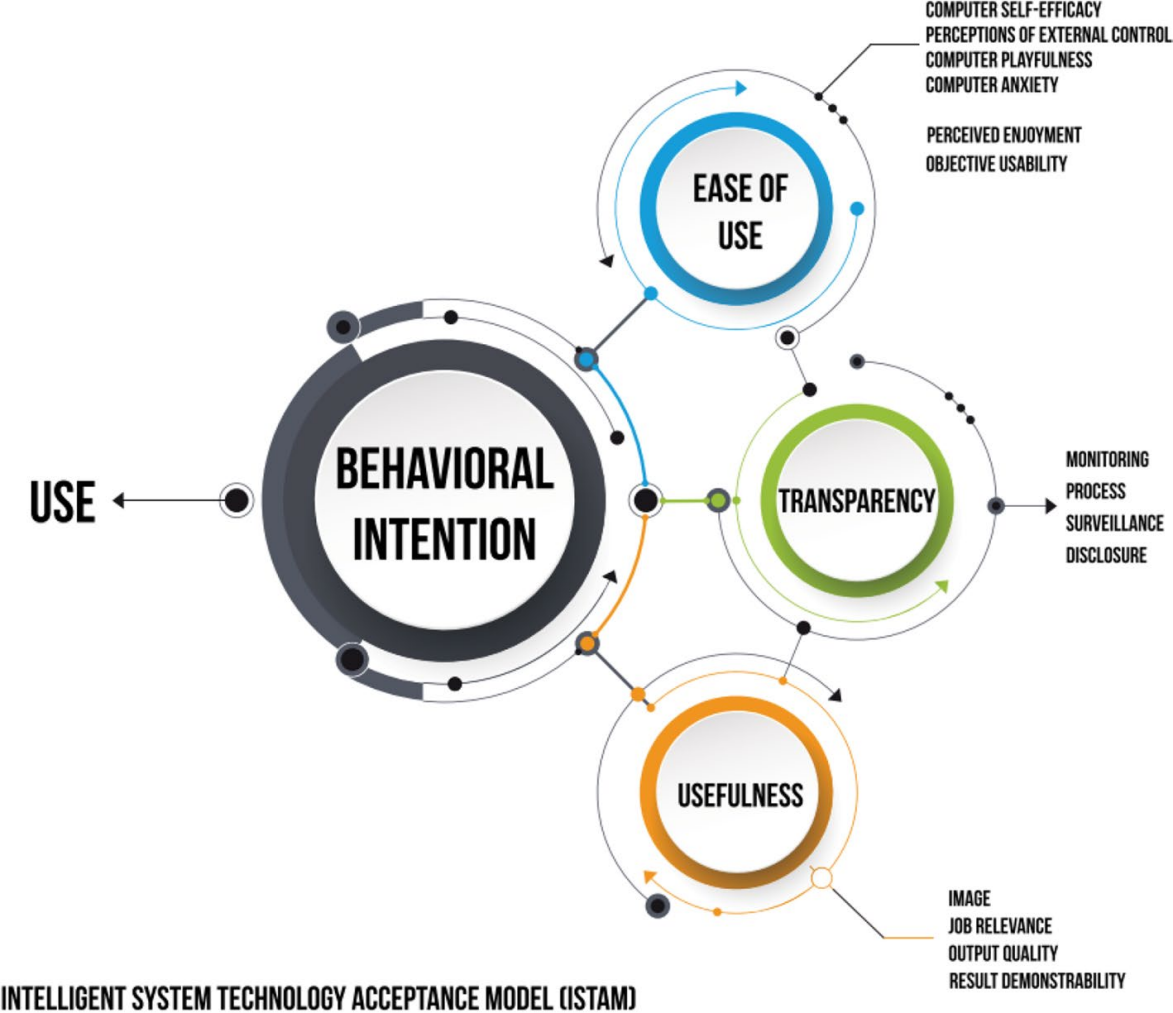
The Intelligent Systems Technology Acceptance Model (ISTAM)

Transcripts were uploaded to N-Vivo (QSR, version 14.23.2) to assist with analysis via an inductive and deductive framework approach [27, 28]. The five main stages of the approach were followed, starting with the lead researcher (SW) familiarizing herself with the data by reading and rereading the transcripts. Initial codes were generated inductively by creating concepts in the data without preconceptions [29]. Codes with shared meanings were grouped into themes, forming an initial framework that was applied across the complete dataset. Data were charted into a matrix (row [themes], sub-row [sub-theme, i.e., codes], column [relevant quote]) to summarize the data [27, 28]. Themes were refined using the constant comparative technique to move back and forth between the data, comparing and contracting themes and evolving explanations until a fit was made [30]. The lead researcher (SW) mapped these themes to a preexisting framework, the Intelligent Systems Technology Acceptance Model (ISTAM) [31] to support the refinement of key themes [32]. ISTAM is an expansion of the technology acceptance model (TAM) that incorporates aspects such as transparency, which affect users’ acceptance of intelligent systems (e.g., AI, robotics, machine learning) (Fig. 2) [31]. Apparent ‘negative cases’ were further examined to refine explanations [32]. Discussions with coauthors (SW, EB, CT, SPS, and RMA) and the wider CODEC-II research team (RM, SB, TW) were conducted at several stages throughout the analytical process.

Data collection continued until data saturation was reached at 21 interviews. Data saturation is the point at which no new data relevant to the research question occurs in subsequent interviews [33]. Achieving data saturation ensures that the data captures the diversity, depth, and nuances of the factors affecting acceptance of using wearable technologies to support the early detection of dementia-causing diseases [33].

#### Rigour

The Standards for Reporting Qualitative Research (SRQR) [34] were followed, and the Consolidated Criteria for Reporting Qualitative Research (COREQ) checklist was completed [35].

## Results

### Participants

Twenty-one CODEC-II participants volunteered, of whom fourteen participants were female and seven male, ranging in age from 42 to 82 years old. Nine participants had SCD, eight had MCI, one was living with dementia with Lewy bodies (DLB), and three were caregivers (Table 2). Most participants identified as white British (*n* = 19), with one participant identifying as white other and one as black Caribbean. Eleven participants reached the UK-mandated level of education (secondary school level), whereas 10 had received higher education (i.e., university level).

**Table 2:** Participant demographics

Participant ID and cognitive diagnosis (year of diagnosis)	Activate smart-phone app schedule	Highest educational attainment	Gender
P1 SCD* (2022)	28-day	Secondary school	Female
P2 SCD (2022)	28-day	Secondary school	Female
P3 MCI** (2018)	14-day	Higher education	Male
P6 SCD (2022)	28-day	Higher education	Male
P7 MCI (2021)	14-day	Higher education	Male
P8 SCD (2022)	28-day	Higher education	Male
P9 MCI (2020)	28-day	Higher education	Female
P10 SCD (2022)	28-day	Secondary school	Female
P11 SCD (2023)	28-day	Higher education	Female
P12 MCI (2022)	14-day	Secondary school	Female
P13 Caregiver (No cognitive impairment)	14-day	Secondary school	Female
P14 MCI (2022)	14-day	Secondary school	Female
P15 DLB*** (2018)	14-day	Secondary school	Male
P16 MCI (2020)	14-day	Secondary school	Male
P17 SCD (2023)	14-day	Secondary school	Male
P18 MCI (2023)	14-day	Secondary school	Female
P19 Caregiver (No cognitive impairment)	28-day	Higher education	Female
P20 MCI (2020)	28-day	Higher education	Female
P21 Caregiver (No cognitive impairment)	28-day	Higher education	Female
P22 SCD (2023)	28-day	Higher education	Female
P24 SCD (2019)	28-day	Secondary school	Female

^*^SCD subjective cognitive decline, **MCI Mild cognitive impairment, ***DLB Dementia with Lewy bodies

All participants used the activity tracker and EEG headband for two weeks. Two participants were either unable to use the passive smartphone app because their smartphones were incompatible with the app (P1, F, person with SCD) or did not feel comfortable using it (P10, F, person with SCD). Twelve participants were randomly allocated to the 28-day schedule on the active smartphone app, whereas nine participants were allocated to the 14-day schedule.

### Key findings

**Fig. 3:**
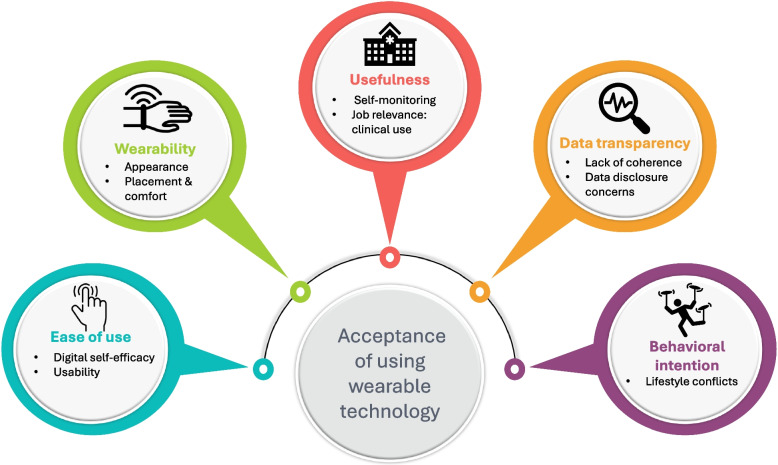
Key themes that were found to affect users'acceptance of wearable technology to support the early detection of dementia-causing diseases

We identified several factors that affected users'acceptance of using wearable technology (Fig. 3). Key themes and subthemes are described below using direct quotations from interviews.

### Ease of use

#### Digital self-efficacy

Many participants felt that they were *“not computer literate at all”* (P13, F, caregiver) and were *“a little bit wary of going into the apps because I didn’t want to upset any of the settings or anything*” (P3, M, living with MCI)*.* Some participants appeared to have low digital self-efficacy (belief in their capacity and confidence in using digital technologies), describing how they were reluctant to use technology within their everyday lives because they did not have “*the patience for it*” (P17, M, living with MCI). They described how important it was to have the support of the research team with the initial onboarding process because *“when it [technology] doesn’t work, I don’t know what to do with it”* (P6, M, person with SCD), but *“once it’s up and running I’m all right”* (P13, F, caregiver). Another participant described how support at the onboarding stage provided reassurance that the technology was set up correctly; they would rather not “*fiddle with it [the technology], and then find out three days later it’s not working because I did something wrong*” (P2, F, person with SCD). Only a few participants were confident in using technology and described how they “*could probably have done it [set up the research study technologies] myself”* (P21, F, caregiver) without any support from the research team.

#### Usability

Many participants thought that the technologies could not be used effectively by those with visual impairments, hearing impairments, mobility issues, or living with cognitive decline. Some participants found the activity tracker screen hard to see when scrolling through the watch display, with one explaining how *“I can’t see unless I put my glasses on, and I haven’t bothered to put my glasses on*” (P24, F, person with SCD). Others found the headband audio recording “*very quiet. You have to almost have it to your ear to hear it*” (P21, F, caregiver). A caregiver described some concerns about the headband, explaining how her mum was “*quite weak, […] she can't hold things very well. […] trying to hold it [headband] in a position and concentrate on pressing the button is [was] hard for her”* (P21, F, caregiver). Participants with cognitive impairments struggled with the memory recall games within the active smartphone app, as they felt it increased their awareness of their decreased cognitive ability, which some found frustrating.

“*Brought it to the forefront that I am forgetting things, I am getting confused, […] when I was doing the tests at times, I was getting quite frustrated.”* (P3, M, living with MCI).

Some compatibility issues between the research study technologies and the participants’ own devices appeared to emerge. Participants found pairing new wearables with their existing smartphones was difficult due to connectivity issues (e.g., lack of access to Wi-Fi); this was felt to have contributed to many participants stopping their use of the technology, as they perceived it “*a waste of time”* and a *“source of frustration because my life is very much on the go, I always have to make sure [that] the Wi-Fi at the place I’m at, is connected*” (P14, F, living with MCI). Other participants noticed that their phones were not compatible with all the games in the active smartphone app, particularly a game that required a gyroscope to detect tilting motion (unlikely to be included in older phone models).

### Wearability

#### Appearance

Participants were conscious of their appearance when wearing the headband, particularly female participants, with one remarking how their “*husband just laughs, obviously every time I get into bed […] I’ve got my headset [headband] and he just looks at me and goes, night*” (P24, F, person with SCD). Some participants with long hair described how the headband *“makes my hair really flat. So, every night you sleep in it you’ve got to dampen your hair and blow dry and fluff it up a bit, which isn’t great […], it’s annoying.”* (P14, F, living with MCI).

#### Placement of the wearable & comfort

Many found the wrist-worn device (activity tracker) the most comfortable device to wear and appeared familiar with its use, describing it as “*basically a watch”* (P11, F, person with SCD). A small number reported irritation due to fastening the device too tightly or a possible allergic reaction to the strap material. Many participants felt that the headband was *“a bit strange the first night or so* […] *you’re just aware something’s there*” (P9, F, living with MCI). This unease appeared to make it harder for participants to sleep, with some removing the headband during the night. A few participants also reported waking up “*with a splitting headache”* (P8, M, person with SCD), which they attributed to *“just physically having something around your [their] head*” (P8, M, person with SCD). Some participants also felt that the instructions that they had to follow to use the headband were quite challenging:

“*These two little lugs [prongs on the headband to stabilize the band] at the back, they rather make your neck sore, and you’ve got to make sure there's no hair under them. That’s a nuisance because I could put a scarf round to keep the hair off, but that slips off*” (P2, F, person with SCD).

Another participant struggled with placing the headband correctly on their head, describing how they wore the headband one night “*slightly lower […], when you lay on your ear, that hurts*” (P12, F, living with MCI). Another participant appeared to improvise by putting the headband “*over the top of everything”* (P3, M, living with MCI) to help ensure a tighter fit. However, participants also described becoming accustomed to wearing the headband over time but felt that two weeks was “*more than enough. I don’t think you could do any more than that. […] I’ll be glad to get this off and have a proper night’s sleep*” (P10, F, person with SCD).

### Usefulness

#### Self-monitoring

The push notifications (set as a manufacture default on the activity tracker) were designed to encourage users to be more physically active and were well liked by some. One participant described how this functionality was *“good for me, […] to give me a little push to move more*” (P10, F, person with SCD). Others used the activity tracker to monitor other aspects of their health, such as “*getting my resting heart rate and everything. Then calories and steps and movement. I found that really useful*” (P15, M, living with DLB). Step count did not appear to interest other participants, with one explaining how she was “*forever walking about doing things*” (P19, F, caregiver). Another participant perceived the activity tracker as less useful, explaining how she did not “*need another device in my life to tell me what I can and can’t do*” (P14, F, living with MCI). This participant was already using a commercially available activity tracker (one that was not provided by the study team) that delivered behavioral prompts, which may have contributed to this sense of being overwhelmed with notifications and reduced perceived autonomy.

#### Job relevance: clinical usefulness

Some participants thought that the data collected by the technology could support more efficient clinical care. One participant described how her doctor had recommended that she use a portable electrocardiogram (ECG) machine at home, and when she noticed her heart rate was too low, she:

*“Just simply went to the stuff, printed out the last two weeks, wrote a letter to the GP on the Monday, Tuesday night, she was on the phone reducing my medication. You can’t get more efficient than that*” (P22, F, person with SCD).

However, some participants preferred meeting with their healthcare professional in person and having the opportunity to discuss their situation with them, explaining how “*otherwise you can’t really ask any questions*” (P9, F, living with MCI). Other participants were concerned that the technology might not capture the holistic information needed to support a diagnosis, with one participant sharing the belief that this could only be done by a healthcare professional “*actually talking to them [patients] and watching how they move and how they react and what their face is doing and those kind of things.*” (P8, M, person with SCD).

A few participants also observed discrepancies between the activity data (steps) that were collected by the study device (activity tracker) and the activity data (steps) collected by their own devices, with one participant explaining:

“*The watch [their personal one] on one hand and the [study activity tracker] on the other, and the watch says I've done twelve thousand steps, and the [study activity tracker name] says sixteen thousand steps. […] It can’t be accurate*” (P2, F, person with SCD).

Others perceived discrepancies in the sleep data collected by the headband in terms of the duration of sleep, describing *“I went to bed about nine o’clock at night and I woke at six o’clock in the morning, yet it [headband] told me I’d had three-and-a-half-hour sleep*” (P15, M, living with DLB). As well as stages of sleep when compared with another study device (activity tracker); “*the [study activity tracker name] will tell me maybe I’ve had 50 min deep [sleep], and then the headset [headband] will tell me I’ve done an hour and a half or two hours [of deep sleep]”* (P24, F, person with SCD).

### Transparency

#### Lack of coherence

A lack of transparency on what data had been collected from wearable technologies appeared to make some participants anxious. They questioned whether their personal information, such as bank account passwords, might have been collected from the passive smartphone app. One participant who shared these concerns *“didn’t want that one [passive smartphone app on their phone] because I've got various banks and stuff like that on my phone”* (P10, F, person with SCD). Other participants posed questions about the safety of the headband and whether wearing it to obtain the EEG “*recording would have a long-term harmful effect*” (P3, M, living with MCI).

#### Data disclosure concerns

Some participants were concerned about whether the health-related data generated from the technology were shared with other entities. One participant wondered if the government might sell their health data to third-party companies, raising *“the whole question of how our data is being used and what it’s being used for”* (P16, M, living with MCI). The majority believed that health-related data were “*not the sort of information I can see being hacked for malicious purposes. Whereas finance, one’s payment processes, ins and outs, clearly is a target for criminality.”* (P6, M, person with SCD).

### Behavioral intention

#### Lifestyle conflicts

Participants reflected on how their daily routine and responsibilities influenced the use of the technologies. Retired participants, without cognitive impairment or with subjective cognitive impairment, commonly reflected on *“one of the beauties of being retired is we can do something like this without a struggle, I just fit it in. So it isn’t even a chore”* (P22, F, person with SCD), while participants with work or volunteering commitments or greater cognitive impairments noted how it was more challenging to integrate these technologies into their lifestyles. For instance, one of the games within the active smartphone app was “*a little bit time-restricting if I'm going to work, because it says, there’s one that says it could be seven minutes long. Well, it may take you longer*” (P10, F, person with SCD). Others found that having a set time to do the game on the active smartphone every day or within a fixed time period was inconvenient, especially if it clashed with a work- or volunteering related meeting, as they would “*quiet[en] it [the reminder] and then think I’ll do that when I’ve finished, and I forgot”* (P15, M, living with DLB).

## Discussion

This study identified several factors affecting the acceptability of wearable technologies for the early detection of dementia-causing diseases among users. These included ease of use, wearability, usefulness, data transparency and behavioral intention. Participants expressed barriers related to the usability of wearables for individuals with busy lifestyles, low digital self-efficacy, visual and hearing impairments, mobility issues, and/or cognitive impairment. Participants preferred wearables that could be worn in a familiar, unobtrusive place, such as the wrist. A lack of transparency about what data had been collected from these wearables and whether it was going to be shared with other entities appeared to make some participants anxious.

Inclusive technology design is vital for the successful adoption of digital technology within healthcare to ensure equitable access, regardless of sociodemographic background [18, 36]. However, current technologies often exclude individuals due to age-related factors, such as declining dexterity, mobility, eyesight, and drier finger skin which can reduce the inbuilt adaptability or functionality of touch screen technologies [37–39]. These barriers affect the reliability, validity, robustness, and clinical usefulness of technologies for older adults [39], who are at risk of developing dementia-causing diseases [40], The design of cognitive testing games in smartphone applications may deter engagement from those with cognitive impairments, as they became more aware of their cognitive impairments, leading to frustration and reduced motivation to use the application. By taking an inclusive-by-design approach, such as involving the user throughout the design and development phase, their needs can be met, and government regulations regarding accessible design standards can be followed [41, 42]. This can proactively address various issues, such as technology abandonment (users stop using a device), while enhancing acceptance of these technologies [43–47]. An individual’s digital self-efficacy and requirement(s) for support must also be considered and addressed to improve inclusivity, particularly during the onboarding process. The need to provide digital support has been recognized by various governments, with the Italian government building a ‘digital academy’ that disseminates educational material to improve the accessibility of digital solutions [48, 49]. The Belgium Government allocated €450 million to the design and implementation of digital literacy projects [49, 50]. The European Union (EU) digital competence framework [51] was produced to guide the development of curricula to equip older adults with essential basic digital skills [52]. In the UK, organisations such as the ‘Centre for Aging Better’ and the ‘Good Things Foundation’ have published good practice principles for delivering digital support to older adults [43]. These eight principles include tailoring the pace of teaching to an individual, creating space for repetition and reflection to consolidate learning, using simple language and avoiding technical terms, and codesigning services with users [43]. These frameworks and principles should be integrated into the design of digital health research to ensure that the user is sufficiently supported in using the technology.

It is important to consider the wearability of the technology, including where the device is worn on the body, as this can affect the social acceptability of the wearable device, especially considering gestures and touches the user may make to the device [53]. Our study highlighted how individuals prefer wearable technology placed on the wrist rather than the head, which is consistent with previous research findings [53–55]. This is likely due to the increased familiarity and understanding of smartwatches [56, 57]. Another key element of wearability that our study highlighted was the comfort an individual experiences when the technology was worn correctly versus incorrectly, highlighting the importance of providing adequate support and guidance to caregivers of individuals who cannot report their discomfort to aid proper use of the technology. For example, checking for skin changes (reddening or dryness) could help reduce harm to vulnerable groups.

Trust and transparency also influenced the acceptance of wearable technologies for health purposes. Similar findings were reported in another study in which surveyed patients (n = 416) reported that trust and privacy concerns, perceived usefulness and perceived ease of use influenced their acceptance of digital health technology [58]. Trust, in particular, has been suggested to play an important role in the successful adoption of digital health technology in the clinical setting, and without it, there is a greater risk of devices not being used (which contributes to increasing costs), increasing user concerns and reducing acceptance of these devices [59, 60]. Strategies are needed to improve communication around technology's limitations, capabilities, and data disclosure. This would help improve the clarity, navigability, and noticeability of the information provided to reassure the user and support their understanding [61]. Some strategies have included using a human-centered design [62], simple lay term language to improve readability for all users, avoiding vague terms that can leave an individual questioning the meaning of phrases, increasing the use of visualizations, meaningfully organizing the text (avoiding a ‘wall of text’ and scattered information around different documents), and considering the length of information as excessively lengthy documents are likely to cause information overload [61]. However, the feasibility of implementing such strategies is questionable due to the amount of resources needed in terms of man hours, technical skill, and time and funding to involve under-served groups (e.g., those with low educational attainment) in human centered design approaches [63].

The final key finding from this study related to unintended behavior changes, such as increasing activity levels, that some individuals reported when using wearable technologies. Previous research has shown various commercially available activity trackers, such as the one used in this study, contain several behavioral change techniques including positive reinforcing messages when physical activity is carried out, proven to increase physical activity in older adults [64]. However, one systematic review suggested wrist worn activity trackers cannot significantly improve physiology (e.g., reducing high blood pressure) [65]. As a higher activity level is known to be a modifiable protective lifestyle factor in reducing the risk of developing a dementia-causing disease [66], and high blood pressure increases an individual’s risk of developing dementia causing diseases [67], further research is needed to explore the impact the use of wrist worn activity trackers may have on the ability to support early detection.

Furthermore, this study highlighted the need to provide users with busy lifestyles with additional support and encouragement to aid in the adoption of technologies. This could be achieved by using behavior change frameworks, such as the COM-B (Capability, Opportunity, and Motivation) model, which support the understanding of why a specific behavior occurs and how to create targeted interventions that lead to effective change [68], The COM-B model could be integrated into the design, development and implementation of wearable technologies in healthcare to promote desired behaviors (i.e., technology adoption). The amount of effort needed from users to adopt the technology should also be minimal. We found that many participants in this study did not have any issues with continuing to use the passive smartphone app after the study had completed but struggled to use the active smartphone app continuously every day for two weeks.

## Recommendations

**Fig. 4:**
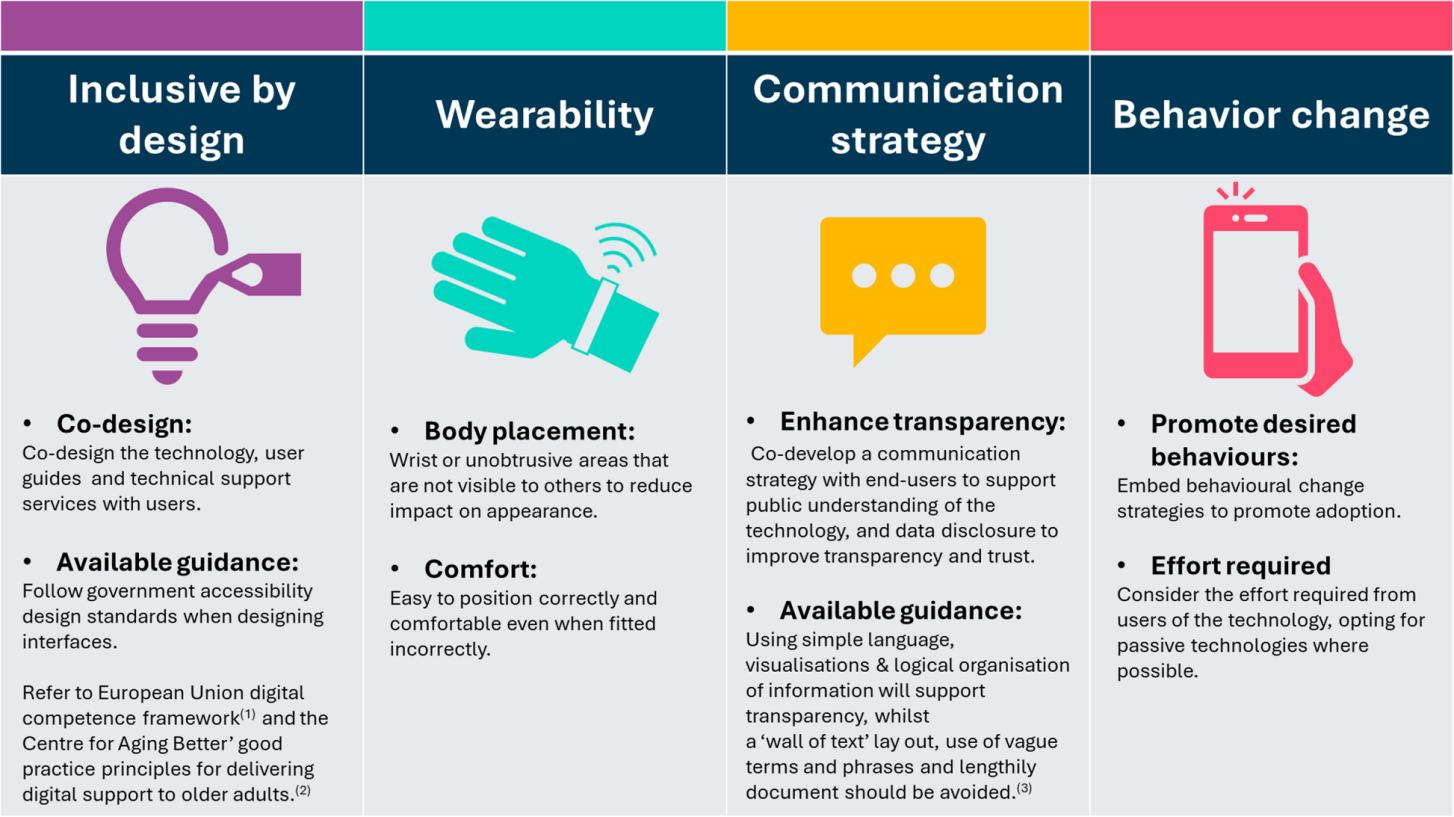
Recommendations for designing, developing, and implementing acceptable digital technologies to support the early detection of dementia-causing diseases

Building on the existing literature and the key findings from this qualitative study, we propose four novel practical recommendations need to be considered by researchers and technology developers when designing, developing, and implementing digital technologies to support the early detection of dementia-causing diseases (Fig. 4).

The first recommendation is to follow an inclusive by design approach. This involves codesigning the technology with the user or, if commercially available technology is used, then all the user guidance and additional health-related information relating to the technologies should be codesigned with the user. Accessibility design standards [41, 42] must also be followed, and digital support services need to be codesigned with users, alongside guidance from the EU digital competence framework [51] and the Centre for Aging Better’s eight good practice principles for delivering digital support to older adults [43]. We also recommend that the government and public health policy and regulatory bodies set recommendations and guidelines for delivering digital support for older adults to improve the standardization of digital support services.

The second recommendation is to consider the wearability of the device, opting for wrist placement or other unobtrusive areas that are not visible to others, to reduce the impact on appearance. It also needs to be easy to position correctly and comfortable (even when fitted incorrectly) to reduce unintended harm among vulnerable groups.

The third recommendation is to codevelop a communication plan with users to increase understanding of the technology and data transparency; this could include the use of visualizations and organizing information in a meaningful, logical manner [61].

The final recommendation is to consider the effort required from users of the technology, opting for passive technologies where possible, as well as embedding behavioral change strategies to promote adoption.

## Strengths and limitations

Strengths of this qualitative study include the collection of data until data saturation was achieved, peer-debriefing was conducted during analysis with various members of the research team, and that all aspects of the research were conducted and reported with a high level of transparency, ensuring rigour and trustworthiness of our findings [34, 35]. Our use of the framework approach, which supports an inductive analysis followed by deductive analysis, aided the examination and refinement of existing technology acceptance theories. However, this study only included a sub-sample of participants from one cohort based in Essex who may hold different views compared to individuals residing in other areas of the UK. For example, the North East of England has the highest prevalence of individuals with low levels of digital engagement (32%) [69], low digital competency (28%) [70], and offline individuals (i.e., those who do not use internet enabled devices or internet based communication routes or information sources (5% in 2023 vs England’s average of 3.6%) [70]. Within certain areas of the North East, older adults in particular, are extremely likely to face digital inequities as 94% of digitally excluded residents in North Tyneside were over 60 years old [71]. Thus, individuals residing in a region with low digital engagement and digital competencies may hold different opinions and perspectives on the use of technology to support the early detection of dementia causing diseases. Furthermore, this study excluded those who could not speak English, did not have a smartphone or internet access and lacked representation of underserved groups who are known to be at risk of digital exclusion, such as individuals without a fixed address or from ethnic minority groups [72]. The use of remote interviews may have unintentionally excluded those with low digital literacy skills and/or those without access to adequate connectivity to support the use of technologies in such a manner, as some participants with access to slow connectivity (Wi-Fi) bandwidth faced buffering issues when using zoom. Mitigating digital exclusion is vital when promoting inclusivity of digital health technologies, as failing to do so can exacerbate health inequities, with underserved groups facing additional barriers when using, accessing, and/or feeling motivated to use digital technology for healthcare purposes [73]. Future work should explore the feasibility and effectiveness of implementing current recommendations to support digital health equity among underserved groups at risk of digital exclusion, such as adopting a collaborative working approach with all users and providers to ensure needs are met and promote inclusivity [8, 72].

## Conclusion

This study identified five key themes that affected the acceptability of wearable technologies to support the early detection of dementia-causing diseases and proposed four novel practical recommendations to support researchers in designing, developing, and implementing digital health interventions in an acceptable, inclusive manner. Future research is needed to assess the effectiveness of implementing recommendations to support digital health equity.

## Supplementary Information

Supplementary Material 1.

## Data Availability

The datasets generated and analyzed during this study are not publicly available due to a potential breach of participant confidentiality. Anonymized datasets may be available from the corresponding author upon reasonable request.

## Abbreviations

AD: Alzheimer’s Disease
CODEC: Predictors of COgnitive DECline using digital devices
COM-B: Capability, Opportunity, and Motivation model
COREQ: Consolidated Criteria for Reporting Qualitative Research
DLB: Dementia with Lewy Bodies
EDoN: Early Detection of Neurodegenerative Diseases
ISTAM: Intelligent Systems Technology Acceptance Model
MCI: Mild Cognitive Impairment
SCD: Subjective Cognitive Decline
SRQR: Standards for Reporting Qualitative Research
UK: United Kingdom
