# Supplementary material for: Acceptability of wearable technology for the early detection of dementia-causing diseases: perspectives from the CODEC II cohort
Source: BMC Digit Health. 2025 Aug 29;3(1):55. doi: 10.1186/s44247-025-00191-3 (PMC12394386; doi:10.1186/s44247-025-00191-3)
Supplement: Supplementary file 1 — Supplementary Material 1. [file 44247_2025_191_MOESM1_ESM.docx]

**Initial interview guide**

What would you prefer I call you?

I would like to start by thanking you for making time to take part in today’s interview. I’m Sarah Wilson, a research assistant and PhD student at Newcastle university. I also work with a team based in Essex which includes Rachel, who you might have met.

I’m interested in understanding your experience of the wearable technology and the apps we asked you to use.  This could be used to aid our research project, which is looking into ways to detect conditions such as dementia earlier than is currently possible.

Are you still okay to talk about your experience with the tech?

Before we start, is it okay to record our conversation? Everything you say will be kept confidential.

To start with…

- Have you been diagnosed with either MCI or dementia?

*If so which one/ do you know the type?*

*do you know the approximate month and year this diagnosis was given*?

Type?

*How do you think your memory and thinking has been since the diagnosis?*

- Are you able to do everyday activities such as making a cuppa or going shopping by yourself?

Now I’ll ask a few questions about your use of technology, your initial thoughts on the devices we sent you and the apps we asked you to install…

1. How often do you usually use technology on a normal day to day basis?

*What bits of tech do you own?*

1. What were your initial views when you first seen the technology?
2. How did you find the set-up process of all the devices?

*How did you go about setting up the devices?*

- *If Rachel helped then ask if they feel they could have done it them self with a set up manual such as a booklet or a video?*
- *Do you feel any like any additional support would have been helpful during set up?*
- *Opinions on setting up by them self*

*How did you find using the login details we provided you, such as the emails and passwords?*

*Did you experience any issues with Bluetooth pairing for the headband or watch?*

*Ask about any devices that they didn’t mention.*

1. How have you been getting on with the devices so far?

*Likes/dislikes*

*Anything troublesome i.e. comfort, size of the font, size of the screens/buttons*

*Have you had any technical issues such as wifi access or been a bit stuck on how something works?*

*Have you used anything else to help the set up process or that has help you use the tech since set up?*

*Ask about any tech they didn’t mention*

1. What are your opinions on the devices now that you’ve used them for a while? Has it changed form the initial few days?

*e.g., Perceived ease of use, benefits of using the system, perceived value or potential of these tools for understanding their own brain health and ageing?*

*Is there any particular devices that you prefer*?

*If the devices gave cross over data (i*.e*. fitbit and dreem give sleep data) did you look at the comparisons between the data it shown*

1. Do you have any concerns about any risks of using the device?

*Particularly around data security (if they used longevity specifically ask about this as issues were raised before)*

*If so what made you feel concerned? (if you can try to tell them how their data is safe)*

1. Do you think your behaviours or health has changed as a result of using the device over the course of the last 2 weeks?

- *Do you think any health conditions have affected the use of the tech?*

1. If these devices were part of a standard health check that are normally provided for certain age categories would you be willing to use them?

*if they say all devices would be too much ask how many they would be willing to use and which ones*

*if they say yes or no ask them why*

1. Do you think you will carry on using the devices?

- Is the duration of use acceptable?

1. Is there any other feedback you’d like to provide or any questions for me?
